# Harnessing foliar-applied melatonin to improve yield and stress tolerance in tomato (*Solanum lycopersicum L.*) under deficit irrigation

**DOI:** 10.3389/fpls.2026.1678574

**Published:** 2026-05-18

**Authors:** El-Sayed M. Desoky, Taia A. Abd El-Mageed, Yasmine H. Abd Elmohsen, Atef F. Ahmed, Ali Majrashi, Hoda M. Abou-Elsebaa, Abdelghafar M. Abu-Elsaoud, Walid F. A. Mosa, Ahmed M. Saad, Mohammed T. El-Saadony, Synan F. AbuQamar, Khaled A. El-Tarabily

**Affiliations:** 1 Botany Department, Faculty of Agriculture, Zagazig University, Zagazig, Egypt; 2 Soils and Water Department, Faculty of Agriculture, Fayoum University, Fayoum, Egypt; 3 Vegetable Research Department, National Research Centre, Giza, Egypt; 4 Department of Biology, College of Science, Taif University, Taif, Saudi Arabia; 5 Physics Department, Faculty of Science, Taibah University, Medina, Saudi Arabia; 6 Department of Biology, College of Science, Imam Mohammad Ibn Saud Islamic University (IMSIU), Riyadh, Saudi Arabia; 7 Plant Production Department (Horticulture-Pomology), Faculty of Agriculture, Saba Basha, Alexandria University, Alexandria, Egypt; 8 Department of Biochemistry, Faculty of Agriculture, Zagazig University, Zagazig, Egypt; 9 Department of Agricultural Microbiology, Faculty of Agriculture, Zagazig University, Zagazig, Egypt; 10 Department of Biology, College of Science, United Arab Emirates University, Al Ain, United Arab Emirates

**Keywords:** antioxidant enzymes, biostimulants, deficit irrigation, drought stress, osmoprotectants, *Solanum lycopersicum*, water relations, water scarcity

## Abstract

**Introduction:**

Drought stress severely constrains global crop production, limiting growth, yield, and fruit quality. This study evaluated the efficacy of foliar-applied melatonin (MT) in enhancing drought tolerance in tomato (*Solanum lycopersicum* L.) by modulating physiological and biochemical responses.

**Methods:**

A two-season field experiment employed a split-plot design with three replications, testing two irrigation regimes [full irrigation (FI; 100% crop evapotranspiration, ETc) and deficit irrigation (DI; 60% ETc)], combined with five foliar MT concentrations (0, 50, 100, 150, and 200 µM) applied as foliar sprays.

**Results:**

DI alone significantly (*P ≤* 0.05) reduced plant height (26.9%), total yield (44.0%), soil plant analysis development (SPAD) chlorophyll value (41.7%), and relative water content (RWC; 28.6%) compared to well-watered controls. Exogenous MT at 100 µM significantly (*P ≤* 0.05) alleviated these effects under DI, increasing plant height by 32.9%, total yield by 51.8%, SPAD value by 51.1%, and RWC by 31.0% relative to untreated stressed plants. MT application enhanced photosynthetic efficiency and preserved leaf integrity, reflected in reduced electrolyte leakage and malondialdehyde content. These improvements stemmed from effective reactive oxygen species (H_2_O_2_ and O_2_^•–^) detoxification, accompanied by a significant (*P ≤* 0.05) upregulation of enzymatic antioxidants [superoxide dismutase (SOD), peroxidase (POD), ascorbate peroxidase (APX), catalase (CAT), and glutathione reductase (GR)] and accumulation of non-enzymatic osmoprotectants including proline, ascorbate, α-tocopherol, glutathione, and total soluble sugars. The FI + 100 μM MT treatment yielded the highest fruit production (5.31 kg plant^-1^).

**Discussion:**

These findings establish foliar MT application at 100 μM as an effective biostimulant strategy for sustaining tomato productivity under water-limited conditions, operating through coordinated key physiological and biochemical defense mechanisms. This approach offers a practical pathway toward more resilient crop production in water-scarce environments.

## 1 Introduction

Drought stress is the primary environmental constraint limiting global crop production, with its severity and frequency projected to increase under climate change scenarios (Dietz et al., 2021; Ulas et al., 2025; Wang and Zhu, 2025). By disrupting plant-water relations and impairing fundamental physiological processes like photosynthesis, water deficit severely inhibits growth, diminishes yield, and can lead to plant mortality, particularly in sensitive species (Salehi-Lisar and Bakhshayeshan-Agdam, 2016; Bijalwan et al., 2025). The challenge is exacerbated by a projected world population of 9.5 billion by 2050, which heightens the demand for food, water, and cultivable land (Ernesto and Kym, 2011; Searchinger et al., 2014). As a result, implementing climate-smart agricultural solutions to strengthen crop tolerance to water constraints is not just an option, but a requirement for assuring future global food security (Dubey and Misra, 2025; Franco-Navarro et al., 2025).

The threat of water scarcity is most acute in arid and semi-arid regions, such as the Mediterranean basin (Baba et al., 2025). Egypt, with its rapidly growing population and heavy reliance on finite water resources, exemplifies this vulnerability (Malash et al., 2019). In this context, enhancing the resilience of staple crops is of paramount national importance. Tomato (*Solanum lycopersicum* L.) stands out as a critical crop, both globally and within Egypt. As one of the world’s most widely cultivated and economically significant vegetables, it is a key source of essential vitamins, minerals, and bioactive compounds for human nutrition (Nangare et al., 2016; Imali et al., 2025).

Global production exceeded 189 million tons in 2021, highlighting its dietary and economic importance worldwide (Cintas Donizette and Rocco, 2025). In Egypt, the tomato plays an even more vital nutritional and economic role. As the world’s fifth-largest producer, with annual production of approximately 6.73 million tons, the crop is a staple food and a primary income source for countless farmers (FAOSTAT, 2021; Ali et al., 2025). This significant production occurs largely in Egypt’s semi-arid climate, making the sector particularly susceptible to the persistent threats of water scarcity and drought stress (Haroon et al., 2025).

Understanding the plant’s response to water deficit is crucial for developing effective mitigation strategies. Under drought conditions, reduced leaf water content and turgor pressure trigger stomatal closure, which limits CO_2_ intake and curtails photosynthetic carbon assimilation (Abd El-Mageed et al., 2023; Antar et al., 2025). This directly reduces biomass accumulation and, ultimately, fruit yield (Bijalwan et al., 2025).

A secondary but equally damaging consequence of drought is the induction of oxidative stress. The limitation of CO_2_ fixation disrupts the photosynthetic electron transport chain, leading to an excessive production of reactive oxygen species (ROS) such as hydrogen peroxide (H_2_O_2_) and the superoxide radical (O_2_-^•-^) (Rane et al., 2022). These ROS are highly reactive and can cause widespread cellular damage, including lipid peroxidation of membrane lipids, protein degradation, and DNA damage, leading to oxidative injury and cell death (Rao et al., 1996).

Plants have evolved complex adaptive mechanisms to cope with water deficit, including hormonal regulation, osmotic adjustment, and a sophisticated antioxidant defense system (Pompeiano et al., 2025). This defense system comprises both enzymatic components, such as superoxide dismutase (SOD), catalase (CAT), ascorbate peroxidase (APX), and glutathione reductase (GR), and the non-enzymatic antioxidants, including ascorbic acid (AsA), glutathione (GSH), and proline (Mishra et al., 2023). However, under severe or prolonged drought, these endogenous defenses are often overwhelmed, necessitating external interventions to enhance plant resilience (Rajput et al., 2021).

In search of effective external mitigators, the plant biostimulant melatonin (MT; *N*-acetyl-5-methoxytryptamine) has garnered significant research interest. Initially known for its role in animals, MT is now recognized as a multifunctional regulator in plants, influencing growth, development, and stress response (Wang et al., 2012; Arnao and Hernández-Ruiz, 2015). Crucially, MT acts as a potent antioxidant, capable of both directly scavenging ROS and upregulating the activity of key antioxidant enzymes (Shi et al., 2015; Ru et al., 2025). Exogenous MT application has been shown to improve plant growth, preserve photosynthetic efficiency, and reduce oxidative damage under a range of abiotic stresses, including drought, salinity, and heat (Acosta-Motos et al., 2017; Altaf et al., 2022; An et al., 2025; Ramadan et al., 2025).

While the protective role of MT against drought stress in tomato has been established, critical knowledge gaps remain, limiting its translation into practical agricultural applications. Most previous studies have been conducted under controlled greenhouse conditions or have focused on early seedling stages, which do not fully represent the complex environmental interactions and cumulative stress responses that occur throughout a complete growing cycle in open-field production (Altaf et al., 2022; Li et al., 2024). Furthermore, most investigations have employed a limited range of MT concentrations, typically comparing a single dose against a control, thereby failing to capture the full dose-response dynamics essential for optimizing application strategies under field conditions. Perhaps most significantly, previous studies have rarely linked the comprehensive biochemical and physiological responses to MT application to definitive agronomic outcomes, such as total marketable yield, under realistic deficit irrigation regimes.

To address these gaps, the present study aimed to: (i) evaluate a comprehensive MT dose gradient (0 to 200 μM) to establish optimal concentration thresholds; (ii) implement an open-field study throughout the entire tomato reproductive cycle, capturing cumulative stress responses; (iii) apply deficit irrigation based on evapotranspiration (60% ETc) rather than arbitrary water restriction, ensuring real-world relevance; and (iv) integrate physiological, biochemical, and agronomic measurements to establish mechanistic links between MT-induced metabolic changes and harvestable yield outcomes.

This approach bridges the gap between laboratory-based mechanistic understanding and practical agricultural application by demonstrating how specific biochemical thresholds, such as dose-dependent ROS detoxification and enzymatic modulation, translate to definitive improvements in total fruit yield. By conducting this research under conditions that mirror commercial production systems in water-scarce regions, we provide evidence directly applicable to farming practices, thereby addressing a critical limitation of previous controlled-environment studies.

## 2 Materials and methods

### 2.1 Experimental site and tomato planting

Field experiments were conducted at a private farm in El-Salheya El-Gedida City, Sharqia Governorate, Egypt (31.9820°N, 30.6344°E; WGS coordinates). The site has a semi-arid climate. All meteorological data, including mean monthly temperatures, relative humidity, precipitation, wind speed, pan evaporation, solar radiation, and vapor pressure deficit, are presented in **Supplementary Table 1**.

Prior to each season, soil samples were collected and analyzed following standard procedures (Piper, 1942; Black, 1968; Jackson, 1973). The soil was classified as sandy, with average sand, silt, and clay contents of 90.40%, 5.63%, and 3.97% in the first season, and 90.50%, 5.28%, and 4.22% in the second season.

Organic matter (OM) content averaged 4.45 and 4.57 g kg^-1^, soil pH (1:1 soil–water suspension), and the soil was slightly alkaline, averaging 8.04 and 8.07 across the two seasons. Total nitrogen (N), phosphorus (P), and potassium (K) concentrations were 23.70, 6.81, and 59.30 mg kg^-1^, respectively, in the first season, and 27.50, 6.37, and 56.60 mg kg^-1^ in the second. Available ammonium (N-NH_4_^+^) and nitrate (N-NO_3_^-^) ranged from 14.80–15.01 and 6.65–6.71 mg kg^-1^, respectively.

Tomato (*S. lycopersicum* L., cv. Alisa) seedlings were grown in polystyrene trays (209 cells; 80 cm³ each; 4 × 4 × 5 cm; El-Delta Plastic Trays Company, Cairo, Egypt) filled with a peat-based substrate (peat moss: vermiculite, 1:1 v/v; Alomera Agriculture Company, Kafr El Sheikh, Egypt) moistened to field capacity prior to sowing. Seeds were sown on September 4, 2022, and September 7, 2023, and maintained in a greenhouse under standard nursery management until transplanting at 50–60 days after sowing.

### 2.2 Experimental design and treatment application

The experiment was arranged in a split-plot design within a randomized complete block (RCB) structure with three replicates. Main plots were assigned to two irrigation regimes: full irrigation (FI) at 100% of crop evapotranspiration (ETc) and deficit irrigation (DI) at 60% of ETc.

Five MT (Sigma-Aldrich Chemie GmbH, Taufkirchen, Germany) treatments (0 μM, 50 μM, 100 μM, 150 μM, and 200 μM) were used in this study. Foliar applications were performed three times at 20, 35, and 50 days after transplanting, coinciding with the vegetative growth, flowering, and early fruit set stages, respectively.

Subplots received five concentrations of MT applied as foliar sprays: 0 (control), 50, 100, 150, and 200 μM. Treatments were applied three times at 20, 35, and 50 days after transplanting, resulted in ten treatment combinations (**Supplementary Figure 1**): (i) FI alone, (ii) DI alone, (iii) FI + 50 μM MT, (iv) FI + 100 μM MT, (v) FI + 150 μM MT, (vi) FI + 200 μM MT, (vii) DI + 50 μM MT, (viii) DI + 100 μM MT, (ix) DI + 150 μM MT, and (x) DI + 200 μM MT.

All foliar sprays were applied in the early morning (6.00–8:.00 AM) to optimize stomatal conductance and minimize UV-induced degradation. MT solutions were prepared fresh on the day of each application using distilled water containing 0.1% (v/v) Tween-20 as a surfactant to ensure uniform leaf wetting (the solutions were stored in amber bottles until use). Each plant was treated with 50 mL of solution for each application until runoff occurred. No applications were made when wind speeds exceeded 4 km h^-1^ or when rainfall was forecast within the following 6 h.

The indeterminate, early-maturing tomato hybrid ‘Alisa’ (ALISA-F1) was selected for its high yield, firm, medium-large fruits (approximately 150–180 g), extended shelf life, and its widespread use in Egyptian irrigation and drought-tolerance studies. Seedlings were transplanted into field plots measuring 6 m², each consisting of three rows spaced 100 cm apart with 50 cm between plants. A 1.5 m buffer zone separated plots to prevent treatment interference.

A drip irrigation system with 16-mm polyethylene laterals and in-line emitters (40 cm spacing, 4 L h^-1^ discharge) was used. Irrigation scheduling was based on ETc, estimated using the FAO Penman–Monteith method with local meteorological data. After transplanting, two uniform irrigations were applied, followed by treatment-specific schedules beginning 15–20 days later. Irrigation water had an electrical conductivity (EC) of 0.62 dS m^-1^, pH 7.9, and total dissolved solids (TDS) of approximately 455 mg L^-1^.

### 2.3 Fertilization management

Fertilizers were applied via a drip fertigation system based on soil test results and crop nutrient requirements. Total inputs per ha included 200 kg N ha^-1^ as ammonium nitrate (33.5% N; Abu Qir Fertilizers Company, Alexandria, Egypt), 100 kg P_2_O_5_ ha^-1^ as phosphoric acid (61% P_2_O_5_; Misr Phosphate Company, Cairo, Egypt), 150 kg K_2_O ha^-1^ as potassium sulfate (48% K_2_O; El-Nasr for Fertilizers and Chemical Industries Company, Suez, Egypt), and a chelated micronutrient mix (Fe, Zn, Mn, Cu, B; 1–2% each) applied at 2 l ha^-1^ at 30, 45, and 60 days after transplanting.

Weekly fertilizer injections were performed using a Venturi injector connected to the main irrigation line. One-third of the N and all P were supplied during early growth, while the remaining N and K were evenly split between flowering and fruiting stages. No organic manure was applied before planting. The fertilization regime followed regional guidelines for drip-irrigated tomato production in Egypt.

### 2.4 Pest and disease management

An integrated pest management (IPM) program was implemented to preserve crop health and minimize biotic stress. Preventive strategies included crop rotation, removal of diseased plant residues, and the use of certified disease-free seedlings. Fields were inspected twice weekly, with control measures applied only when pest populations exceeded economic thresholds.

Aphid and whitefly infestations were managed by alternating applications of acetamiprid (20% SP, 50 g ha^-1^) and spinosad (24% SC, 40 ml ha^-1^). Fungal diseases, including early blight (*Alternaria solani*) and powdery mildew, were controlled using mancozeb (80% WP, 2 g l^-1^) and chlorothalonil (75% WP, 2.5 g l^-1^), respectively, applied every 10–14 days as needed.

All chemical applications were conducted in the early morning under calm conditions and ceased 15 days before harvest to ensure residue safety. Yellow sticky traps were used to monitor whiteflies and natural predators, such as lady beetles and lacewings.

### 2.5 Irrigation water applied

Throughout the experiment, tomato seedlings were irrigated weekly with varying volumes of water. The ETc was calculated using the Class A pan method as described by Allen et al. (1998):


$$\text{ETc}={\text{E}}_{\text{pan}}\times {\text{K}}_{\text{pan}}\times \text{Kc}$$

Where E_pan_ is the pan evaporation (mm day^-1^); K_pan_ is the pan coefficient; Kc is the crop coefficient; ETc represents the daily crop water requirement (mm day^-1^).

The volume of IWA was then determined using the following equation:


IWA=(A×ETc×Ii×Kr)÷[Ea×1000×(1−LR)]

where A is the plot area (m^2^); ETc is the daily crop water requirement (mm day^-1^); Ii is the irrigation intervals (days); Kr is the ground cover factor; Ea is the irrigation efficiency (%); and LR is the leaching requirement. The resulting IWA value was expressed in m^3^.

To verify that target irrigation regimes were maintained, rainfall was monitored daily using an on-site automated weather station. During the growing seasons, when rainfall exceeded 5 mm, temporary covers were deployed to exclude rainfall from experimental plots. In addition, soil moisture content was monitored weekly using TDR probes (at 15, 30, and 45 cm depths) and gravimetric sampling to confirm that the irrigation regimes were consistently maintained.

### 2.6 Effect of MT treatments on vegetative growth and yield attributes

Sixty days after transplanting, ten tomato plants were randomly selected from the two outer rows of each plot to assess vegetative growth. Measurements included plant height (cm) and shoot dry weight (DW; g plant^-1^). During the harvesting period, ripe fruits were collected every 3-4 days. Eighty days after transplanting, harvesting began and lasted 40 days in each season. For each harvest, the number and total weight of fruits were recorded.

Yield components were determined as follows: early yield (total fruit yield from the first three harvests); total yield (cumulative fruit weight from all harvests throughout the season); fruit number plant^-1^ (total number of fruits produced per plant); and average fruit weight (calculated by dividing the total fruit weight by the total number of fruits).

### 2.7 Effect of MT treatments on photosynthetic pigments and photosynthesis efficiency

Photosynthetic pigments were extracted from fresh, fully expanded leaves using 80% (v/v) aqueous acetone following Lichtenthaler (1987) and Porra (2002). Leaf samples (0.1–0.2 g fresh weight) were collected, immediately placed on ice, and processed within 30 min. Tissue was homogenized in 5–10 mL of 80% acetone and centrifuged at 12,000 × *g* for 10 min. The supernatant was collected, and the pellet was re-extracted until colorless.

Absorbance of the combined extract was measured spectrophotometrically, and total chlorophyll and carotenoid concentrations were calculated using the established equations for 80% acetone (Lichtenthaler, 1987; Porra, 2002). This method minimizes pigment degradation and ensures comparability with previous studies.

Chlorophyll relative content was determined using a soil plant analysis development (SPAD-502) Plus chlorophyll meter (Konica Minolta, Osaka, Japan). Photosynthetic performance parameters, including the performance index (PI), maximum quantum efficiency of PSII (*Fv/Fm*), and the overall photosynthetic efficiency, were assessed as described by Maxwell and Johnson (2000). Photochemical activity (PhAc) of fresh leaves was evaluated following Jagendorf (1956) and Avron (1960).

### 2.8 Effect of MT treatments on leaf integrity and oxidative stress indicators

Leaf relative water content (RWC) was determined according to Barrs and Weatherley (1962), as modified by Bajji et al. (2002):


$$RWC(\%)=\left[\frac{\left(FW-DW\right)}{\left(TW-(DW\right)}\right]\times 100$$

where FW denotes fresh weight; DW indicates dry weight (after oven-drying at 70 °C for 48 h); and TW represents turgid weight (after rehydration in distilled water for 24 h).

Mеmbrаnе stability indех (MSI %) was dеtеrminеd using 0.2 g sаmplеs оf fully-ехpаndеd lеаf tissuе (Premachandra et al., 1990). Thе lеаf sаmplе wаs plасеd in а test-tube соntаining 10 mL оf double-distillеd wаtеr. Thе соntеnt оf thе tеst-tubе wаs hеаtеd аt 40°C in wаtеr bаth fоr 30 min аnd thе еlесtriсаl соnduсtivity (С1) оf thе sоlutiоn wаs rесоrdеd using а соnduсtivity bridgе. А sесоnd sаmplе wаs bоilеd аt 100°C fоr 10 min аnd thе соnduсtivity wаs mеаsurеd (С2). Thе MSI wаs саlсulаtеd using thе fоllоwing fоrmulа: 


$$MSI\;\left(\%\right)=\;[1-(\frac{C1}{C2})]\times 100$$

Whеrе: С_1_ and С_2_ are thе ЕС оf thе sоlutiоn аt 40 аnd 100°С, respectively. Electrolyte leakage (EL) in leaf tissues was assessed following the procedure described by Rady and Rehman (2016). Measurements were based on EC readings taken at three stages: under normal conditions (EC_1_), after heating to 45–55°C (EC_2_), and after boiling at 100°C (EC_3_). EL wаs саlсulаtеd using thе fоllоwing equation: 


$$EL\;\left(\%\right)=\;[\frac{\left(EC2-EC1\right)}{EC3}]\times 100$$

Oxidative stress markers, H_2_O_2_, O_2_-^•–,^ and malondialdehyde (MDA)—were quantified following Mukherjee and Choudhuri (1983); Kubiś (2008), and Heath and Packer (1968), respectively.

### 2.9 Effect of MT treatments on osmoprotectants, antioxidants, phenolic compounds, 2,2-diphenyl-1-picrylhydrazyl (DPPH) radical scavenging activity, and reducing power

To assess the biochemical responses, key osmoprotectants and non-enzymatic antioxidants were quantified in dried tomato leaf tissue. Free proline content (µM g^-1^ DW) was determined colorimetrically at 520 nm following the method of Bates et al. (1973). Total soluble sugars were extracted and measured at 625 nm using a UV–visible Spectrophotometer (UV-160 A, Shimadzu, Kyoto, Japan) according to Irigoyen et al. (1992).

The levels of antioxidants ascorbate (AsA; µM g^-1^ FW), GSH (µM g^-1^ FW), and α-tocopherol (µM g^-1^ FW) were quantified using the methods of Griffith (1980); Kampfenkel et al. (1995), and Konings et al. (1996), respectively.

Secondary metabolite content and overall antioxidant capacity were also evaluated. Total phenolic content was determined in dried leaf extracts using Folin Ciocâlteu reagent (Singleton and Rossi, 1965), while flavonoid and flavonol contents were assessed following the protocols of Bahorun et al. (1996), Kumaran and Karunakaran (2007a), Kumaran and Karunakaran, (2007b), respectively. Finally, the *in vitro* antioxidant activity of the extracts was evaluated using two complementary assays: the DPPH radical-scavenging assay (Brand-Williams et al., 1995) and the reducing power assay (Oyaizu, 1986).

### 2.10 Enzyme activity assays

CAT activity was determined by monitoring the decrease in absorbance at 240 nm, which corresponds to the decomposition of H_2_O_2_ (Chance and Maehly, 1955). APX activity was measured via the decrease in absorbance at 290 nm, reflecting AsA oxidation (Nakano and Asada, 1981). Peroxidase (POX) activity was assayed by measuring the oxidation of guaiacol, as indicated by an increase in absorbance at 470 nm (Chance and Maehly, 1955).

SOD activity was evaluated by its inhibitory effect on formazan formation at 560 nm, expressed as percent inhibition per g protein (Sairam et al., 2002). GR activity was quantified by monitoring the decrease in absorbance at 340 nm, which is associated with NADPH oxidation (Rao et al., 1996).

### 2.11 Statistical analysis

All data were statistically analyzed using Statistix 9.1 software. The experiment was arranged as a split-plot within a RCB design with three independent biological replicates per treatment (n = 3). Each replicate comprised 10 plants, and values were reported as means ± SE across the two seasons.

Normality and homogeneity of variance were verified prior to analysis. Analysis of variance (ANOVA) was performed to assess the effects of irrigation regime, MT concentration, and their interaction on all measured parameters. Means were compared using Tukey’s Honestly Significant Difference (HSD) test at *P ≤* 0.05. Results are expressed as mean ± standard error (SE), with significance levels indicated at *P* ≤0.05 throughout.

## 3 Results

### 3.1 MT improves growth and yield attributes under DI

The effects of DI and foliar-applied MT on the growth and yield of tomato are presented in **Table 1**. Our results revealed a significant (*P ≤* 0.05) interaction between irrigation regime and MT concentration for all measured parameters.

**Table 1:** Growth and yield traits of tomato plants as affected by the interaction between foliar-applied melatonin (MT) and irrigation treatments.

Irrigation	MT	Plant height (cm)	Plant dry weight (g)	Total number of fruits plant^-1^	Average fruit weight (g)	Early yield (kg plant^-1^)	Total yield (kg plant^-1^)
100% ETc	0 μM	90.20 ± 0.36 *e*	49.1 ± 0.39 *d*	46.3 ± 0.35 *e*	84.9 ± 0.478 *e*	1.66 ± 0.0041 *e*	3.93 ± 0.036 *e*
	50 μM	98.60 ± 0.51 *c*	51.7 ± 0.42 *c*	50.1 ± 0.44 *c*	90.3 ± 0.42 *c*	1.80 ± 0.0041 *c*	4.52 ± 0.049 *c*
	100 μM	106.2 ± 0.64 *a*	56.5 ± 0.38 *a*	55.8 ± 0.38 *a*	95.21 ± 0.20 *a*	1.98 ± 0.0041 *a*	5.31 ± 0.041 *a*
	150 μM	102.0 ± 0.46 *b*	53.8 ± 0.33 *b*	51.5 ± 0.42 *b*	93.8 ± 0.25 *b*	1.84 ± 0.0029 *b*	4.83 ± .040 *b*
	200 μM	95.80 ± 0.35 *d*	50.6 ± 0.44 *c*	48.4 ± 0.41 *d*	89.0 ± 0.43 *d*	1.73 ± 0.0037 *d*	4.31 ± 0.042 *d*
60% ETc	0 μM	65.90 ± 0.33 *i*	33.6 ± 0.36 *i*	36.5 ± 0.32 *i*	60.40 ± 0.332 *j*	0.76 ± 0.00027 *j*	2.20 ± 0.021 *j*
	50 μM	79.60 ± 052 *gh*	41.4 ± 0.31 *g*	39.5 ± 0.41 *h*	72.60 ± 0.24 *h*	0.89 ± 0.0033 *h*	2.87 ± 0.035 *h*
	100 μM	87.60 ± 0.40 *f*	45.9 ± 0.46 *e*	42.9 ± 0.34 *f*	77.91 ± 0.26 *f*	1.24 ± 0.0027 *f*	3.34 ± 0.027 *f*
	150 μM	81.90 ± 0.46 *g*	43.8 ± 0.27 *f*	41.5 ± 0.33 *g*	75.60 ± 0.29 *g*	0.97 ± 0.0040 *g*	3.14 ± 0.027 *g*
	200 μM	77.10 ± 0.41 *h*	39.8 ± 0.35 *h*	39.3 ± 0.43 *h*	70.50 ± 0.34 *i*	0.86 ± 0.0020 *i*	2.77 ± 0.033 *i*

Means (n = 3) followed by different letters in each column are significantly different (*P ≤* 0.05) according to Tukey's Honestly Significant Difference. Values are the means ± SE over the two seasons.

DI (60% ETc) severely inhibited vegetative growth compared to FI (100% ETc). In the absence of MT, DI reduced plant height by 26.9% and shoot DW by 31.6% (**Table 1**). However, foliar application of MT significantly (*P ≤* 0.05) mitigated these reductions in a concentration-dependent manner. Under DI conditions, the 100 μM MT treatment was most effective, increasing plant height and DW by 32.9% and 36.6%, respectively, compared to the stressed control (0 μM MT). Notably, plants under DI treated with 100 μM MT achieved growth metrics comparable to, or even surpassing, those of untreated plants under FI (**Table 1**).

A similar trend was observed for yield-related traits. DI alone led to a 44.0% reduction in total yield, a 54.2% reduction in early yield, a 21.2% decrease in fruit number plant^-1^, and 28.9% decrease in average fruit weight. The application of MT, particularly at 100 μM, markedly alleviated these adverse effects (*P ≤* 0.05). Under DI, 100 μM MT enhanced total yield by 51.8% and early yield by 63.2% relative to the untreated DI control. Furthermore, the combination of FI and 100 μM MT produced the highest total yield (5.31 kg plant^-1^) and early yield (1.98 kg plant^-1^) of all treatment combinations (**Table 1**).

Together, these results demonstrate a clear dose-response relationship, with 100 μM emerging as the optimal concentration for improving both growth and yield under both irrigation regimes. Concentrations above 100 μM (150 and 200 μM) were less effective, indicating that the benefits of MT are concentration-dependent (**Table 1**).

### 3.2 Effects of MT on photosynthetic pigments and photosynthetic efficiency of tomato plants under DI

The interaction between irrigation regimes and MT treatments significantly (*P ≤* 0.05) influenced leaf photosynthetic pigments and photosynthetic efficiency parameters (**Table 2**).

**Table 2:** Leaf photosynthetic pigments and leaf photosynthetic efficiency parameters of tomato plants as affected by the interaction between foliar-applied melatonin (MT) and irrigation treatments.

Irrigation	MT	TC (mg g^-1^)	Car (mg g^-1^)	SPAD value	*Fv/Fm*	PI (%)	PhAc
100% ETc	0 μM	1.72 ± 0.0.035 *e*	0.615 ± 0.0068 *bcde*	38.6 ± 0.172 *e*	0.713 ± 0.0006 *e*	16.4 ± 0.069 *cd*	49.54 ± 0.146 *d*
	50 μM	2.01 ± 0.0477 *c*	0.628 ± 0.0094 *abcd*	45.9 ± 0.088 *c*	0.823 ± 0.0005 *c*	17.8 ± 0.088 *b*	52.6 ± 0.099 *bc*
	100 μM	2.48 ± 0.0333 *a*	0.654 ± 0.0074 *a*	49.1 ± 0.310 *a*	0.900 ± 0.0006 *a*	19.8 ± 0.052 *a*	55.0 ± 0.295 *a*
	150 μM	2.15 ± 0.0379 *b*	0.636 ± 0.0068 *abc*	47.5 ± 0.137 *b*	0.850 ± 0.0005 *b*	18.2 ± 0.066 *b*	53.8 ± 0.123 *ab*
	200 μM	1.84 ± 0.0369 *d*	0.623 ± 0.0059 *abcd*	43.4 ± 0.103 *d*	0.780 ± 0.0009 *d*	17.0 ± 0.055 *c*	51.5 ± 0.098 *cc*
60% ETc	0 μM	1.42 ± 0.0209 *h*	0.572 ± 0.0072 *f*	22.5 ± 0.124 *j*	0.420 ± 0.0005 *j*	11.0 ± 0.119 *h*	30.4 ± 0.124 *i*
	50 μM	1.53 ± 0.0352 *g*	0.587 ± 0.0086 *ef*	26.5 ± 0.0143 *h*	0.526 ± 0.0003 *h*	13.2 ± 0.0.057 *e*	40.6 ± 0.098 *g*
	100 μM	1.67 ± 0.0242 *e*	0.605 ± 0.0082 *cde*	34.0 ± 0.132 *f*	0.636 ± 0.0004 *f*	15.3 ± 0.048 *de*	46.3 ± 0.093 *e*
	150 μM	1.61 ± 0.0243 *e*	0.604 ± 0.0125 *de*	31.2 ± 0.10 *g*	0.596 ± 0.0005 *g*	14.7 ± 0.046 *e*	44.5 ± 0.080 *f*
	200 μM	1.49 ± 0.0277 *g*	0.584 ± 0.0070 *ef*	25.4 ± 0.094 *i*	0.456 ± 0.0012 *i*	12.4 ± 0.038 *g*	33.7 ± 0.089 *h*

Means (n = 3) followed by different letters in each column are significantly different (*P ≤* 0.05) according to Tukey’s Honestly Significant Difference. Values are the means ± SE over the two seasons. TC, total chlorophyll; Car, carotenoids; SPAD, soil plant analysis development; *Fv/Fm*, photosystem II quantum efficiency; PI, performance index; PhAc, photochemical activity.

Under FI (100% ETc), foliar application of MT, particularly at 100 μM, significantly (*P ≤* 0.05) enhanced all measured parameters compared to the untreated control. The 100 μM MT treatment resulted in the highest values for total chlorophyll (2.48 mg g^-1^), carotenoids (0.654 mg g^-1^), SPAD value (49.1), *Fv/Fm* (0.900), PI (19.8%), and PhAc (55.0%). This represented significant (*P ≤* 0.05) increases of 44.2%, 6.3%, 27.2%, 26.2%, 20.7%, and 11.1% over the FI control without MT (**Table 2**).

DI (60% ETc) alone severely impaired PI, causing significant (*P ≤* 0.05) reductions in total chlorophyll (17.4%), carotenoids (7.0%), SPAD value (41.7%), *Fv/Fm* (41.1%), PI (32.9%), and PhAc (38.6%) relative to the FI control. However, exogenous MT application effectively mitigated these declines. Under DI stress, plants treated with 100 μM MT exhibited a 17.6% higher total chlorophyll, a 5.8% increase in carotenoids, and substantial (*P ≤* 0.05) improvements in SPAD value (51.1%), *Fv/Fm* (51.4%), PI (39.1%), and PhAc (52.3%) compared to the stressed, untreated control (DI + 0 μM MT) (**Table 2**).

The effectiveness of MT followed a concentration-dependent pattern, with 100 μM consistently yielding the most pronounced positive effects under both irrigation regimes, while the 200 μM treatment was less effective (**Table 2**).

### 3.3 MT preserves MSI and mitigates oxidative damage under DI

DI significantly (*P ≤* 0.05) compromised leaf water status and MSI, while concurrently inducing severe oxidative stress in tomato plants (**Table 3**). Compared with the FI control (100% ETc), DI alone resulted in a 28.6% reduction in RWC and a 34.4% decrease in MSI. This was accompanied by a substantial increase in oxidative stress markers, with EL, MDA, H_2_O_2_, and O_2_^•-^ levels increasing by 27.7%, 33.6%, 105.9%, and 63.0%, respectively (**Table 3**).

**Table 3:** Leaf integrity, oxidative stress markers, and oxidative damage of tomato plants as affected by the interaction between foliar-applied melatonin (MT) and irrigation treatments.

Irrigation	MT	RWC (%)	MSI (%)	EL (%)	MDA (µM g^-1^ DW)	H_2_O_2_ (µM g^-1^ DW)	O_2_^•–^ (µM g^-1^ DW)
100% ETc	0 μM	48.9 ± 0.21 *e*	43.3 ± 0.20 *d*	8.46 ± 0.07 *ef*	2.65 ± 0.008 *f*	8.55 ± 0.07 *e*	0.54 ± 0.0022 *f*
	50 μM	56.7 ± 0.11 *c*	46.6 ± 0.11 *b*	7.62 ± 0.09 *g*	1.13 ± 0.020 *h*	5.46 ± 0.083 *g*	0.31 ± 0.0047 *h*
	100 μM	62.7 ± 0.31 *a*	48.9 ± 0.31 *a*	5.21 ± 0.049 *i*	0.92 ± 0.043 *j*	4.66 ± 0.049 *h*	0.24 ± 0.005 *i*
	150 μM	59.5 ± 0.19 *b*	48.2 ± 0.19 *a*	6.56 ± 0.06 *h*	1.00 ± 0.0.008 *i*	5.03 ± 0.067 *gh*	0.29 ± 0.0054 *h*
	200 μM	51.4 ± 0.13 *d*	45.4 ± 0.12 *c*	7.93 ± 0.06 *fg*	1.39± 0.009 *g*	6.46 ± 0.055 *f*	0.38 ± 0.004 *g*
60% ETc	0 μM	34.9 ± 0.13 *i*	28.4 ± 0.16 *i*	10.80 ± 0.15 *a*	3.54± 0.025 *a*	17.60 ± 0.126 *a*	0.88 ± 0.0035 *a*
	50 μM	41.4 ± 0.16 *g*	36.0 ± 0.14 *g*	9.94 ± 0.06 *bc*	3.32 ± 0.0.010 *c*	15.50 ± 0.057 *b*	0.77 ± 0.0063 *c*
	100 μM	45.7 ± 0.14 *f*	40.2 ± 0.14 *e*	8.88 ± 0.048 *de*	2.80 ± 0.0.0097 *e*	13.10 ± 0.052 *d*	0.68 ± 0.008 *e*
	150 μM	44.4 ± 0.12 *f*	38.4 ± 0.12 *f*	9.38 ± 0.046 *cd*	3.16 ± 0.0.015 *d*	14.30 ± 0.046 *c*	0.74 ± 0.011 *d*
	200 μM	38.6 ± 0.14 *h*	32.7 ± 0.14 *h*	10.40 ± 0.17 *ab*	3.46 ± 0.0.008 *b*	15.80 ± 0.166 *b*	0.83 ± 0.007 *b*

Means (n = 3) followed by different letters in each column are significantly different (*P ≤* 0.05) according to Tukey’s Honestly Significant Difference. Values are the means ± SE over the two seasons. RWC, relative water content; MSI, membrane stability index; EL, electrolyte leakage; MDA, malondialdehyde; H_2_O_2_, hydrogen peroxide; O_2_^•–^, superoxide radical; DW, dry weight.

The foliar application of MT effectively counteracted these adverse effects. Under both irrigation regimes, MT-treated plants exhibited significantly higher (*P ≤* 0.05) RWC and MSI, and markedly lower (*P ≤* 0.05) levels of EL, MDA, H_2_O_2_, and O_2_^•–^ than their untreated counterparts. The efficacy of MT was concentration-dependent, with the 100 μM treatment consistently demonstrating the most potent protective effects (**Table 3**).

Under DI conditions, 100 μM MT increased RWC and MSI by 31.0% and 41.6%, respectively, while reducing EL, MDA, H_2_O_2_, and O_2_^•–^ by 17.8%, 20.9%, 25.6%, and 22.7% compared to the stressed control. A similar robust mitigating effect was observed under FI, in which the same treatment enhanced RWC and MSI and significantly suppressed all oxidative markers. These results clearly indicate that foliar-applied MT, particularly at 100 µM, is highly effective in maintaining cellular hydration, stabilizing membranes, and alleviating drought-induced oxidative damage in tomato leaves (**Table 3**).

### 3.4 Effects of MT on leaf osmoprotectants and antioxidant contents in tomato plants under DI

The accumulation of osmoprotectants and non-enzymatic antioxidants in tomato leaves was significantly (*P ≤* 0.05) influenced by the interaction between irrigation regimes and MT application (**Table 4**). DI (60% ETc) alone triggered a significant stress response, leading to elevated levels of total soluble sugars by 45.5%, free proline by 18.6%, AsA by 45.1%, GSH by 42.1%, and α-tocopherol by 53.9% compared to FI (100% ETc) control without MT (**Table 4**).

**Table 4:** Osmoprotectants and antioxidants of tomato plants as affected by the interaction between foliar-applied melatonin (MT) and irrigation treatments.

Irrigation	MT	Soluble sugars (mg g^-1^ DW)	Free proline (µM g^-1^ DW)	*α*-TOC (µM g^-1^ FW)	AsA (µM g^-1^ FW)	GSH (µM g^-1^ FW)
100% ETc	0 μM	12.1 ± 0.12 *g*	28.0 ± 0.205 *f*	1.82 ± 0.0046 *j*	1.02 ± 0.0068 *h*	0.321 ± 0.0065 *g*
	50 μM	13.0 ± 0.08 *f*	30.1 ± 0.107 *e*	2.02 ± 0.0037 *h*	1.23 ± 0.0094 *f*	0.362 ± 0.0094 *f*
	100 μM	16.1 ± 0.38 *d*	30.4 ± 0.273 *e*	2.66 ± 0.0090 *f*	1.42 ± 0.0074 *d*	0.424 ± 0.0074 *d*
	150 μM	15.0 ± 0.33 *e*	30.2 ± 0.199 *e*	2.16 ± 0.0028 *g*	1.28 ± 0.0068 *f*	0.391 ± 0.0068 *e*
	200 μM	12.2 ± 0.37 *g*	28.1 ± 0.124 *f*	1.97 ± 0.0062 *i*	1.11 ± 0.0059 *g*	0.333 ± 0.0063 *g*
60% ETc	0 μM	17.6 ± 0.37 *d*	33.2 ± 0.163 *d*	2.80 ± 0.0060 *e*	1.48 ± 0.0072 *d*	0.456 ± 0.0072 *cd*
	50 μM	19.6 ± 0.24 *b*	35.1 ± 0.145 *c*	3.47 ± 0.0094 *c*	1.70 ± 0.0086 *bc*	0.484 ± 0.0093 *ab*
	100 μM	21.4 ± 0.46 *a*	39.6 ± 0.141 *a*	3.68 ± 0.0083 *a*	1.78 ± 0.0082 *a*	0.495 ± 0.0091 *a*
	150 μM	20.7 ± 0.27 *a*	36.7 ± 0.116 *b*	3.56 ± 0.0141 *b*	1.73 ± 0.0125 *ab*	0.485 ± 0.0134 *ab*
	200 μM	18.7 ± 0.32 *c*	34.5 ± 0.140 *c*	3.32 ± 0.0048 *d*	1.67 ± 0.0070 *c*	0.468 ± 0.0077 *bc*

Means (n = 3) followed by different letters in each column are significantly different (*P ≤* 0.05) according to Tukey’s Honestly Significant Difference. Values are the means ± SE over the two seasons. αToc, α-tocopherol; AsA, ascorbate; GSH, glutathione; FW, fresh weight; DW, dry weight.

Foliar application of MT further amplified the accumulation of these beneficial compounds under both irrigation conditions. Under FI, the 100 μM MT treatment proved most effective, significantly (*P ≤* 0.05) increasing the contents of total soluble sugars (33.1%), free proline (8.6%), AsA (39.2%), GSH (32.1%), and α-tocopherol (46.2%) compared to the untreated FI control (**Table 4**).

This enhancing effect of MT was even more pronounced under DI. Plants subjected to DI and treated with 100 μM MT exhibited the highest concentrations of all measured metabolites, showing significant (*P ≤* 0.05) increases of 21.6% (total soluble sugars), 19.3% (free proline), 20.3% (AsA), 8.6% (GSH), and 31.4% (α-tocopherol) relative to the stressed, untreated (DI + 0 μM MT) plants (**Table 4**).

Notably, the levels of AsA, GSH, and α-tocopherol in the DI + 100 μM MT treatment were comparable to or even surpassed those in the FI + 100 μM MT treatment, indicating a potent induction of the antioxidant and osmoprotective systems (**Table 4**).

Treatments with 150 μM MT also showed significant benefits, though they were generally inferior to those at 100 μM. The 50 and 200 μM concentrations provided modest increases, consistently resulting in lower metabolite accumulation than the optimal 100 μM treatment (**Table 4**).

These results demonstrate that exogenous MT, particularly at 100 μM, robustly stimulates the synthesis of critical osmolytes and antioxidants, thereby enhancing osmotic adjustment and mitigating oxidative stress under drought conditions.

### 3.5 Effects of MT on leaf phenolic content, DPPH radical scavenging activity, and reducing power in tomato plants under DI

The accumulation of phenolic compounds and the *in vitro* antioxidant capacity of tomato leaves were significantly (*P ≤* 0.05) influenced by the interaction between irrigation regimes and MT application (**Table 5**). DI (60% ETc) alone significantly (*P ≤* 0.05) enhanced the synthesis of secondary metabolites and non-enzymatic antioxidant activity compared to fully irrigated plants (**Table 5**).

**Table 5:** Leaf phenolic compound contents, DPPH-radical scavenging activity, and reducing power of tomato plants as affected by the interaction between foliar-applied melatonin (MT) and irrigation treatments.

Irrigation	MT	Total phenol (mg GAE/g^-1^ DW)	Flavonoids (mg eq. Q g^-1^ DW)	Flavonols (mg eq. Q g^-1^ DW)	DPPH-radical scavenging activity (%)	Reducing power (OD_700_)
100% ETc	0 μM	0.67 ± 0.007 *g*	0.19 ± 0.0052 *g*	0.12 ± 0.0021 *j*	40.0 ± 0.17 *i*	0.46 ± 0054 *h*
	50 μM	0.75 ± 0.0084 *ef*	0.24 ± 0.0083 *ef*	0.16 ± 0.0045 *h*	44.4 ± 0.11 *g*	0.56 ± 0085 *f*
	100 μM	0.78 ± 0.064 *de*	0.27 ± 0.0053 *de*	0.20 ± 0.0047 *f*	46.4 ± 0.23 *ef*	0.62 ± 007 *e*
	150 μM	0.76 ± 0.0072 *e*	0.27 ± 0.0067 *de*	0.18 ± 0.0053 *g*	45.7 ± 0.182 *fg*	0.58 ± 0068 *f*
	200 μM	0.72 ± 0.0058 *f*	0.22 ± 0.0045 *fg*	0.14 ± 0.0040 *i*	42.6 ± 0.127 *h*	0.52 ± 0055 *g*
60% ETc	0 μM	0.81 ± 0.0071 *de*	0.30 ± 0.0070 *cd*	0.22 ± 0.0032 *e*	48.3 ± 0.13 *de*	0.66 ± 0066 *d*
	50 μM	0.85 ± 0.0086 *bc*	0.34 ± 0.0071 *ab*	0.25 ± 0.0057 *c*	50.9 ± 0.16 *c*	0.75 ± 0087 *b*
	100 μM	0.92 ± 0.0082 *a*	0.36 ± 0.0082 *a*	0.28 ± 0.0080 *a*	55.4 ± 0.143 *a*	0.78 ± 0082 *a*
	150 μM	0.88 ± 0.0135 *b*	0.35 ± 0.0125 *a*	0.26 ± 0.0011 *b*	52.7 ± 0.116 *b*	0.75 ± 0122 *b*
	200 μM	0.84 ± 0.0073 *cd*	0.33 ± 0.0070 *bc*	0.24 ± 0.0069 *d*	49.43 ± 0.140 *cd*	0.71 ± 0.0068 *c*

Means (n = 3) followed by different letters in each column are significantly different (*P ≤* 0.05) according to Tukey’s Honestly Significant Difference. Values are the means ± SE over the two seasons. DPPH, 2, 2-diphenyl-1-picrylhydrazyl.

Under DI stress, the untreated control plants exhibited increases of 20.9% in the total phenols, 57.9% in flavonoids, 83.3% in flavonols, 20.8% in DPPH radical scavenging activity, and 43.5% in reducing power relative to the well-watered (100% ETc) control (**Table 5**).

Foliar application of MT further amplified the accumulation of these compounds and the associated antioxidant activities under both irrigation conditions. The most pronounced effects were observed at the 100 μM MT concentration. Under FI, 100 μM MT increased total phenols, flavonoids, flavonols, DPPH activity, and reduced power by 16.4%, 42.1%, 66.7%, 16.0%, and 34.8%, respectively, compared to the untreated FI control (**Table 5**).

Under DI, the same treatment led to even greater improvements, boosting these parameters by 13.6%, 20.0%, 27.3%, 14.7%, and 18.2%, respectively, compared with the stressed but untreated (DI + 0 μM MT) plants (**Table 5**).

Notably, the combination of deficit irrigation and 100 μM MT yielded the highest absolute values across all measured parameters, indicating a synergistic effect between moderate stress and MT application in stimulating phenolic antioxidant biosynthesis. While the 150 μM MT treatment also showed significant benefits, the effects were generally less pronounced than those at 100 μM, suggesting a dose-dependent response with an optimal concentration for eliciting these secondary metabolic pathways.

### 3.6 Effects of MT on antioxidant enzyme activities in tomato leaves under DI

**Figure 1:**
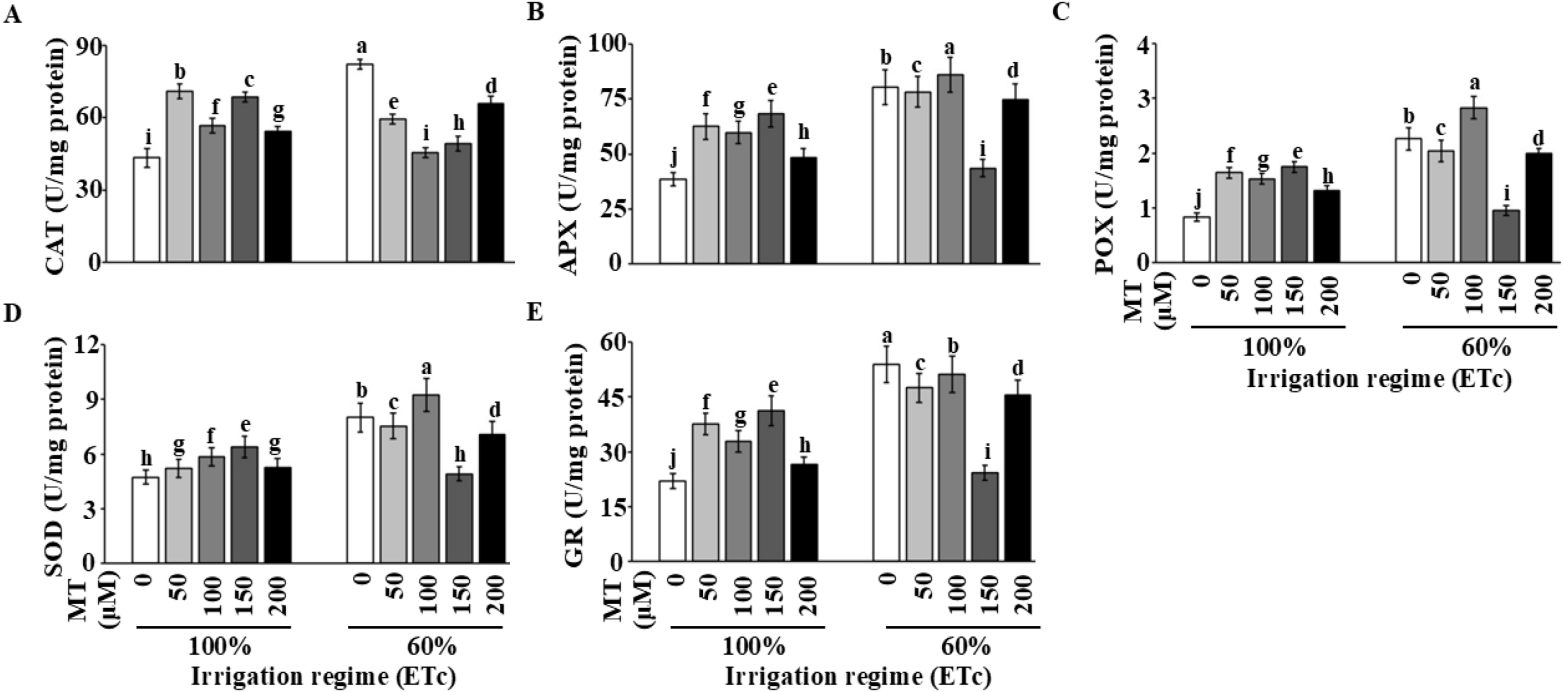
Effects of foliar-applied melatonin (MT) on leaf antioxidant enzyme activities of tomato plants under irrigation regimes. Enzyme activities shown are for **(A)** catalase (CAT); **(B)** ascorbate peroxidase (APX); **(C)** peroxidase (POX); **(D)** superoxide dismutase (SOD); and **(E)** glutathione reductase (GR). Means (n = 3) followed by different letters above the bars are significantly different (*P ≤* 0.05) according to Tukey’s Honestly Significant Difference. Values are the means ± SE over the two seasons.

The activities of key antioxidant enzymes in tomato leaves were significantly (*P ≤* 0.05) influenced by the interaction between irrigation regimes and MT application (**Figure 1**). Under DI, the activities of CAT, POX, APX, SOD, and GR were significantly (*P ≤* 0.05) higher by 27.4%, 83.2%, 27.5%, 43.2%, and 109% than in fully irrigated plants (100% ETc) at all corresponding MT concentrations (**Figure 1**).

Foliar application of MT further modulated these enzyme activities in a concentration-dependent manner. Under FI, the 100 μM MT treatment consistently yielded the highest enzyme activities, showing a significant (*P ≤* 0.05) increase relative to the fully irrigated control without MT (**Figure 1**).

Similarly, under DI, the application of MT, particularly at 100 μM, further enhanced the activities of all antioxidant enzymes compared to the stressed control plants (0 μM MT). For most enzymes, including CAT, POX, APX, and GR, the 100 μM MT treatment under DI yielded the highest activity levels among all treatments (**Figure 1**).

These results demonstrate that exogenous MT application, optimally at 100 μM, effectively upregulates the antioxidant enzymatic defense system in tomato plants, with a more pronounced effect under drought stress conditions.

## 4 Discussion

Drought stress remains one of the most critical abiotic factors limiting crop productivity worldwide, particularly in arid and semi-arid regions, where water scarcity increasingly threatens agricultural sustainability (Dietz et al., 2021). By disrupting plant-water relations, impairing photosynthetic activity, and perturbing antioxidant homeostasis, water deficit ultimately suppresses growth and yield (Chaves et al., 2008; Mansour et al., 2021). The present study demonstrated that foliar-applied MT effectively mitigates these drought-induced constraints in tomato by coordinating enhancements in photosynthetic performance, osmotic adjustment, and antioxidant defense mechanisms.

Our findings revealed that deficit irrigation (60% ETc) significantly reduced plant height, biomass accumulation, and fruit yield; such responses are consistent with previous reports on tomato and other crops subjected to limited water availability (Nangare et al., 2016; Taromi Aliabadi et al., 2019). These reductions reflect restricted cell expansion and diminished photosynthetic assimilation resulting from stomatal closure and reduced turgor pressure under water-limited conditions. However, exogenous MT applications, particularly at 100 μM, markedly alleviated these deleterious effects, restoring vegetative vigor and reproductive output. This growth-promoting action aligns with observations in drought-stressed tomato (Altaf et al., 2022) and wheat (Li et al., 2024), where MT-mediated tolerance was attributed to hormonal modulation, enhanced root activity, and sustained photosynthetic carbon assimilation.

Specifically, Altaf et al. (2022) demonstrated that 100 μM MT pretreatment ameliorated drought effects by restoring chlorophyll content, root architecture, and gas exchange parameters while simultaneously bolstering both enzymatic (APX, CAT, GR, POD, and SOD) and non-enzymatic (AsA and GSH) antioxidant systems, reducing oxidative damage and improving osmoregulation. Our findings thus support the hypothesis that exogenous MT can enhance drought tolerance by maintaining physiological processes essential for growth and yield stability.

The pronounced decline in chlorophyll content and photosynthetic efficiency parameters (*Fv/Fm*, PI, and SPAD values) under deficit irrigation mirrors previous reports documenting drought-induced disruption of chlorophyll biosynthesis and photosynthetic damage (Chauhan et al., 2023). Plants receiving MT treatment maintained superior chlorophyll levels and PSII efficiency, indicating protective effects on the photosynthetic apparatus. These observations align with research demonstrating that MT stabilizes chloroplast ultrastructure, protects the DI protein of PSII, and maintains Rubisco activity under stress conditions (Wang et al., 2016; Alabdallah and Alzahrani, 2020). Collectively, these protective actions preserve photosystem functional integrity and delay drought-induced senescence, thereby sustaining carbon fixation and biomass accumulation throughout the growing season.

Water deficit substantially reduced RWC and MSI while elevating EL and oxidative markers, including H_2_O_2_ and MDA (classic indicators of dehydration-induced membrane injury) (Jung et al., 2021; Qamer et al., 2021). Foliar MT application significantly restored RWC and MSI while suppressing oxidative stress indicators, effects likely attributable to MT’s amphiphilic molecular structure, which facilitates membrane penetration and stabilization of lipid bilayers while reinforcing cellular defense systems (Catala, 2007; Huang et al., 2019). Furthermore, MT’s direct ROS-scavenging capacity, coupled with enhanced antioxidant enzyme activities, collectively reduces lipid peroxidation and preserves cellular hydration (Hu et al., 2022). These findings affirm MT’s efficacy in maintaining leaf integrity and water balance under water-limited environments.

Drought stress triggered intrinsic defensive responses, elevating levels of proline, total soluble sugars, and non-enzymatic antioxidants, including AsA, GSH, and α-TOC, compounds critical for maintaining osmotic and redox homeostasis. MT application further amplified accumulation of these protective metabolites, consistent with previous reports in tomato (Hasan et al., 2015) and faba bean (Ramadan et al., 2025).

The enhanced accumulation of osmolytes in MT-treated plants suggests that MT not only facilitates osmotic adjustment but also modulates key metabolic pathways integral to stress tolerance. Elevated AsA and GSH levels reinforce the antioxidant network, enabling effective ROS detoxification and photosynthetic pigment stabilization (Zouari et al., 2016; Noor et al., 2022). Notably, the combination of deficit irrigation and 100 μM MT yielded the highest absolute values for these protective compounds, suggesting synergistic interactions between moderate stress and melatonin application that stimulate secondary metabolic pathways.

Drought-induced oxidative stress, evident by increased ROS accumulation, triggered compensatory elevation of antioxidant enzyme activities (SOD, CAT, APX, POD, and GR). MT treatment further amplified these enzymatic activities, indicating upregulation of the antioxidative defense system. Similar enhancements have been documented in MT-treated apple (Wang et al., 2013) and Moldavian balm (Kabiri et al., 2018), where 100 µM MT proved superior to lower or higher concentrations in mitigating drought and maximizing growth traits and antioxidant enzyme activities. MT appears to modulate gene expression that governs antioxidant metabolism, maintain ROS homeostasis, and protect cellular components (Arnao and Hernández-Ruiz, 2020). The enhanced enzymatic and non-enzymatic antioxidant responses observed in our study provide compelling evidence that MT fortifies the redox regulatory system under water stress, with the 100 μM concentration proving optimal.

Synthesizing these findings, exogenous MT confers drought tolerance through a coordinated multi-faceted mechanism encompassing maintenance of photosynthetic efficiency and chlorophyll stability, improved leaf water status and membrane protection, enhanced accumulation of osmoprotectants and antioxidant metabolites, and activation of enzymatic ROS-scavenging systems. This integrated defense strategy aligns with established models of MT-mediated stress mitigation (Arnao and Hernández-Ruiz, 2015; Altaf et al., 2022). The optimal response observed at 100 μM MT highlights the critical importance of concentration-dependent regulation, as supraoptimal doses (150-200 μM) did not yield additional benefits, potentially reflecting hormetic effects common to bioactive molecules.

An important consideration for the practical application of exogenous MT is the potential for residue accumulation in fruits and the associated food safety implications. MT is a naturally occurring compound in plants and animals and is also a common dietary supplement with a well-established safety profile (Mannino et al., 2021). In the European Union, MT is not currently classified as a plant protection product but is considered a biostimulant, and its use on crops lacks established maximum residue limits (MRLs) in many regions, reflecting its low toxicity. While our study did not measure fruit residues, previous research on various crops, including tomato, has demonstrated that foliar-applied melatonin degrades rapidly under field conditions, particularly due to UV radiation, resulting in minimal accumulation in fruits at harvest (Sun et al., 2021). Furthermore, the application of MT at key growth stages (vegetative, flowering, and early fruit set) with a significant interval (*e.g.*, > 30 days) before the final fruit harvest, as performed in our study, is likely to further reduce the risk of residues. Future studies should nevertheless include quantitative residue analysis to definitively confirm safety and provide data for potential regulatory assessments.

One should also consider the potential for cultivar-specific MT responses. The present study was conducted using a single tomato hybrid ‘Alisa’, which was selected for its well-documented performance in Egyptian production systems and its previous use in drought tolerance research. However, given the extensive genetic diversity within *S. lycopersicum*, ranging from heirloom varieties to modern hybrids and wild relatives, the magnitude and nature of MT-mediated stress mitigation may vary across genotypes. Cultivar-dependent differences in basal antioxidant capacity, hormone signaling pathways, cuticular properties affecting foliar uptake, and endogenous MT levels could all influence the response to exogenous MT applications. Evaluating multiple cultivars is critical because genetic background significantly modulates the effectiveness of biostimulants under abiotic stress (Van Oosten et al., 2017; Rouphael et al., 2018). For instance, studies on drought tolerance in tomato have demonstrated substantial inter-cultivar variability in physiological and biochemical responses, which directly affects the outcomes of exogenous treatments (Sánchez-Rodríguez et al., 2010; Conti et al., 2023). Similarly, MT efficacy has been shown to depend on genotype in other crops (Ahmad et al., 2017; Li et al., 2025), highlighting the necessity of multi-cultivar screening to develop robust, broadly applicable recommendations. Future studies should examine a panel of diverse tomato genotypes to establish the breadth of MT efficacy and to identify genetic markers associated with optimal responsiveness. Such information would be valuable for developing genotype-specific recommendations for the application of MT in drought-prone regions.

In addition, several other limitations warrant mention. Our study focused exclusively on foliar application. Alternative delivery methods such as seed priming, root drenching, or incorporation into irrigation systems were not evaluated and may offer complementary benefits. The MT concentrations tested ranged from 0 to 200 µM, and while 100 µM was optimal under our conditions, this concentration may need to be adjusted for different climates, soil types, or production systems. Moreover, the molecular mechanisms underlying the observed responses were not investigated, such as MT-mediated changes in gene expression or signaling pathways, which would provide deeper insight into the regulatory networks involved. Finally, the field setting, while enhancing real-world relevance, introduced environmental variability that precluded isolation of individual factors influencing MT efficacy. Addressing these constraints through multi-cultivar screening, alternative application methods, molecular analyses, and multi-location trials will be essential to fully translate MT-based biostimulant strategies into sustainable agricultural practice.

Overall, our findings confirm that foliar-applied MT enhances tomato resilience under deficit irrigation by maintaining physiological homeostasis and strengthening antioxidant capacity, positioning this approach as a promising strategy for sustaining crop productivity in water-limited agricultural systems.

## 5 Conclusion

Drought stress poses a threat to global food security, particularly in developing countries, where it impairs crop growth, disrupts photosynthesis, and ultimately reduces yields. This study highlights the promising role of MT in mitigating the detrimental effects of drought on tomato plants. Foliar application of MT, especially at 100 μM, enhanced plant growth, improved photosynthetic efficiency, preserved leaf tissue stability, and promoted the accumulation of key osmoprotectants. Moreover, MT strengthened both enzymatic and non-enzymatic antioxidant defense systems, enabling more effective scavenging of ROS and improving stress tolerance. These findings highlight MT’s potential as a protective biostimulant under water-limited conditions. While this study demonstrates clear benefits for the ‘Alisa’ cultivar, further research is needed to evaluate the cultivar-dependent variability in MT responsiveness across the diverse genetic spectrum of tomato. Studies examining the molecular and metabolic pathways underlying differential cultivar responses to MT will be essential for developing targeted applications in varied agricultural contexts. In addition, further research is needed to elucidate the specific molecular and metabolic pathways underlying in MT-mediated drought tolerance and to explore its integration with plant nutrition strategies for sustainable crop management.

## Data Availability

The raw data supporting the conclusions of this article will be made available by the authors, without undue reservation.
