# Supplementary material for: Harnessing foliar-applied melatonin to improve yield and stress tolerance in tomato (Solanum lycopersicum L.) under deficit irrigation
Source: Front Plant Sci. 2026 May 18;17:1678574. doi: 10.3389/fpls.2026.1678574 (PMC13222809; doi:10.3389/fpls.2026.1678574)
Supplement: Supplementary file 1 [file DataSheet1.pdf]

Supplementary Materials

Harnessing foliar-applied melatonin to improve yield and stress tolerance in tomato (*Solanum lycopersicum* L.) under deficit irrigation

El-Sayed M. Desoky, Taia A. Abd El-Mageed, Yasmine H. Abd Elmohsen, Atef F. Ahmed, Ali Majrashi, Hoda M. Abou-Elsebaa, Abdelghafar M. Abu-Elsaoud, Walid F.A. Mosa, Ahmed M. Saad, Mohammed T. El-Saadony, Synan F. AbuQamar[*], Khaled A. El-Tarabily[*]

Correspondence

Synan F. AbuQamar (sabuqamar@uaeu.ac.ae)

Khaled A. El-Tarabily (ktarabily@uaeu.ac.ae)

Table S1. Meteorological data of El-Salheya El-Gedida City, Sharqia governorate, Egypt, in 2022-2023 (Season I) and 2023-2024 (Season II).

Month	Season															
	SI (2022/2023)								SII (2023/2024)							
	Temp.		WS	RH	PPT	PE	SR (Rs, MJ m^{-2} day^{-1})	VPD (kPa)	Temp.		WS	RH	PPT	PE	SR (Rs, MJ m^{-2} day^{-1})	VPD, (kPa)
	Max	Min							Max	Min						
	°C		km/h	%	mm/month	mm/day			°C		km/h	%	mm	mm/day		
**November**	**29.4**	**11.5**	**2.12**	**60.5**	**9.0**	**4.57**	**13.98**	**1.24**	**34.1**	**11.1**	**2.36**	**61.3**	**2.40**	**5.57**	**13.45**	**1.148**
**December**	**27.7**	**6.8**	**1.99**	**66.7**	**21.6**	**3.71**	**12.12**	**0.867**	**28.9**	**9.7**	**2.30**	**70.8**	**24.6**	**4.00**	**11.08**	**0.987**
**January**	**22.3**	**2.4**	**2.53**	**66.2**	**29.1**	**2.81**	**11.98**	**0.598**	**26.5**	**6.0**	**2.10**	**69.5**	**39.3**	**3.1**	**12.58**	**0.850**
**February**	**26.3**	**5.5**	**2.34**	**67.9**	**10.8**	**3.2**	**15.25**	**0.921**	**29.3**	**3.7**	**2.37**	**69.1**	**14.4**	**4.1**	**14.75**	**1.164**

Temp., air temperature; WS, wind speed; RH, relative humidity; PPT, precipitation; PE, Pan evaporation; SR, solar radiation; VPD, vapor pressure deficit

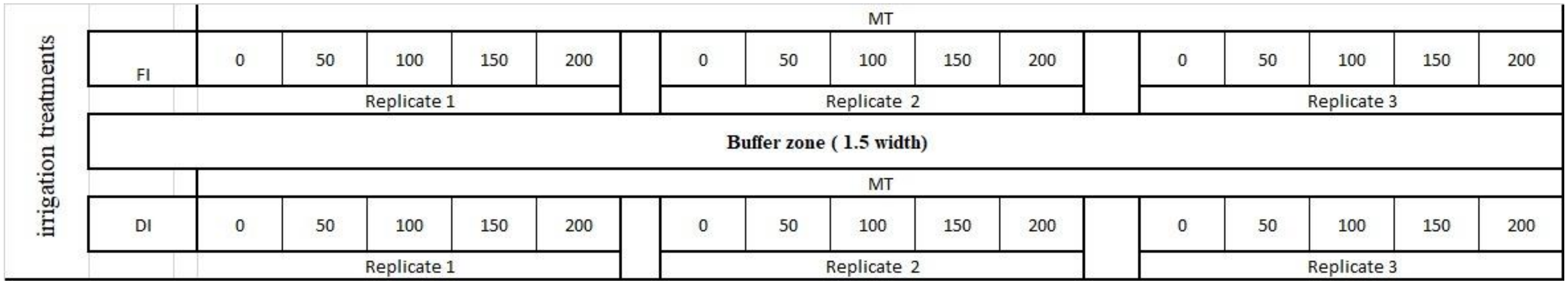

Figure S1. Layout of the field experimental design. Full irrigation (FI; 100% crop evapotranspiration, ETc); deficit irrigation (DI; 60% ETc); MT, foliar melatonin treatment.
